# Precisely mapping a major gene conferring resistance to Hessian fly in bread wheat using genotyping-by-sequencing

**DOI:** 10.1186/s12864-015-1297-7

**Published:** 2015-02-21

**Authors:** Genqiao Li, Ying Wang, Ming-Shun Chen, Erena Edae, Jesse Poland, Edward Akhunov, Shiaoman Chao, Guihua Bai, Brett F Carver, Liuling Yan

**Affiliations:** Department of Plant and Soil Sciences, Oklahoma State University, 368 AG Hall, Stillwater, OK 74078 USA; Hard Winter Wheat Genetics Research Unit, USDA-ARS, Manhattan, KS 66506 USA; Department of Plant Pathology, Kansas State University, Manhattan, KS 66506 USA; United States Department of Agriculture, Agricultural Research Service, Cereal Crops Research Unit, Fargo, ND 58102 USA

**Keywords:** Hessian fly resistance, Genotyping-by-sequencing (GBS), Insect resistance pathway, Quantitative trait loci (QTL), Wheat

## Abstract

**Background:**

One of the reasons hard red winter wheat cultivar ‘Duster’ (PI 644016) is widely grown in the southern Great Plains is that it confers a consistently high level of resistance to biotype GP of Hessian fly (Hf). However, little is known about the genetic mechanism underlying Hf resistance in Duster. This study aimed to unravel complex structures of the Hf region on chromosome 1AS in wheat by using genotyping-by-sequencing (GBS) markers and single nucleotide polymorphism (SNP) markers.

**Results:**

Doubled haploid (DH) lines generated from a cross between two winter wheat cultivars, ‘Duster’ and ‘Billings’ , were used to identify genes in Duster responsible for effective and consistent resistance to Hf. Segregation in reaction of the 282 DH lines to Hf biotype GP fit a one-gene model. The DH population was genotyped using 2,358 markers developed using the GBS approach. A major QTL, explaining 88% of the total phenotypic variation, was mapped to a chromosome region that spanned 178 cM and contained 205 GBS markers plus 1 SSR marker and 1 gene marker, with 0.86 cM per marker in genetic distance. The analyses of GBS marker sequences and further mapping of SSR and gene markers enabled location of the QTL-containing linkage group on the short arm of chromosome 1A. Comparative mapping of the common markers for the gene for *QHf.osu-1A*^*d*^ in Duster and the Hf-resistance gene for *QHf.osu-1A*^*74*^ in cultivar ‘2174’ showed that the two Hf resistance genes are located on the same chromosome arm 1AS, only 11.2 cM apart in genetic distance. The gene at *QHf.osu-1A*^*d*^ in Duster has been delimited within a 2.7 cM region.

**Conclusion:**

Two distinct resistance genes exist on the short arm of chromosome 1A as found in the two hard red winter cultivars, 2174 and Duster. Whereas the Hf resistance gene in 2174 is likely allelic to one or more of the previously mapped resistance genes (*H9*, *H10*, *H11*, *H16*, or *H17*) in wheat, the gene in Duster is novel and confers a more consistent phenotype than 2174 in response to biotype GP infestation in controlled-environment assays.

**Electronic supplementary material:**

The online version of this article (doi:10.1186/s12864-015-1297-7) contains supplementary material, which is available to authorized users.

## Background

Hard red winter wheat (*Triticum aestivum* L, AABBDD genome, 2*n* = 6*x* = 42) is the most widely grown crop in the US Great Plains. Hessian fly [Hf, *Mayetiola destructor* (Say)] is one of the most destructive pests that significantly reduce grain yield and end-use quality of wheat in this area and worldwide [1-4]. Hessian fly is classified into biotypes of A through L, and GP. Biotype GP is the prevalent biotype in fields in the Great Plains area [5,6]. Developing resistant wheat cultivars adapted to this region is the most feasible strategy to minimize losses caused by Hf.

Seven (*H5*, *H9*, *H10*, *H11*, *H16*, *H17*, and *Hdic*) of the 35 resistance genes heretofore identified were reported to reside on the short arm of chromosome 1A and confer resistance against biotype GP [3,7-11], and four of them (*H9*, *H16, H17,* and *Hdic*) also confer resistance against Hf biotype L, the most virulent and prevalent biotype in the eastern USA [1]. All of the seven resistance genes may be arranged as a gene cluster and are reported to exist in tetraploid *T. durum* or *T. dicoccum* or have been transferred from tetraploid wheat to hexaploid wheat [12,13]. In recent studies, efforts have been made to deploy resistance genes that exist in adapted wheat cultivars. A winter wheat cultivar ‘2174’ adapted to the southern Great Plains was found to have a major resistance gene on chromosome 1AS (*QHf.osu-1A*) that confers approximately 70% resistance to biotype GP [4]. A minor QTL on the telomere region of chromosome 1AS in the winter wheat cultivar ‘Clark’ is also associated with resistance to biotype GP [3]. In addition, a minor QTL (*QHf.uga-1AS*) in wheat cultivar AGS 2000 adapted to the eastern USA was reported to confer partial resistance to biotype vH13 [1]. These independent studies have pointed out that chromosome 1AS is a copious resource of effective resistance to multiple biotypes, but it is not known if this region contains multiple resistance genes, or one resistance gene with multiple alleles against Hessian fly, or a combination of both.

The resistance gene at *QHf.osu-1A* in 2174 and its derived cultivars can be immediately utilized to control Hessian fly in winter wheat improvement programs, but the QTL/gene in 2174 explained the majority but not all of the phenotypic variation [4]. This QTL/gene also produces an inconsistent phenotype even under controlled environmental conditions. Novel and more effective sources of Hessian fly resistance in adapted genetic backgrounds are therefore urgently needed in wheat breeding programs. Hard red winter wheat cultivar ‘Duster’ (PI 644016) is now widely grown in the southern Great Plains following its release in 2006, due to its versatility in grain-only and dual-purpose systems and its resilience to biotic and abiotic stress factors. Duster showed the lowest fly intensities among 30 entries tested with moderately to heavily infested Hf in the field for multiple years [14]. Duster is one of a few wheat cultivars that confer a consistently high level of resistance to biotype GP [5]. Moreover, Hf intensities at economically significant levels have not been reported in any field plot containing this unique cultivar [15]. However, little is known about the genetic mechanism underlying Hf resistance in Duster. A diagnostic molecular marker for the resistance gene in Duster is needed for effective resistance breeding.

Recent progress in the application of high-throughput sequencing technologies and development of genomic mapping tools has accelerated identification of agriculturally important genes in QTL mapping experiments [16]. A high-throughput array to interrogate 9,000 gene-associated single-nucleotide polymorphisms (Wheat 9 K iSelect SNP assay) in worldwide accessions of hexaploid wheat including landraces and modern cultivars was developed to detect key genomic regions for wheat improvement [17]. The developed SNP chips and maps of genetic variation have been used to identify new sources of resistance to wheat stem rust, caused by *Puccinia graminis* f. sp. *tritici* race group Ug99, with numerous studies reporting both qualitative genes and quantitative trait loci [18].

More recently, next-generation sequencing (NGS) technology has provided scientists with unprecedented tools to unravel allelic variation associated with complex traits [19]. Several approaches that combine marker discovery and genotyping have been developed, including sequencing of reduced representation libraries, restriction-site-associated DNA sequencing (RAD-seq), multiplexed shotgun sequencing and genotyping-by-sequencing (GBS). Increased output of the GBS data and reduced per sample cost by generating the same amount of data per sample using a 96-plex library have enabled this genotyping platform to become more attractive [20]. The two-enzyme GBS approach has been demonstrated to be robust for genotyping in species with a large and complex genome like barley, and even polyploid genomes like common wheat [20]. The development of high-density GBS markers in hexaploid wheat will facilitate the determination of the physical location of a gene of interest.

In the present work, we employed NGS technology to identify GBS SNP markers across an entire genome for Hf-resistance genotyping of a large wheat population. We have successfully located the gene for resistance within a region of 2.7 cM flanked by two GBS markers. The resistance gene in Duster is also located on the short arm of chromosome 1A, but it is different from the gene previously reported in 2174. The two genes are located 11.2 cM apart in genetic distance. The ability to distinguish these two genes is critically important in marker-assisted breeding.

## Results

### Near immunity of duster to the Hf biotype GP

**Figure 1:**
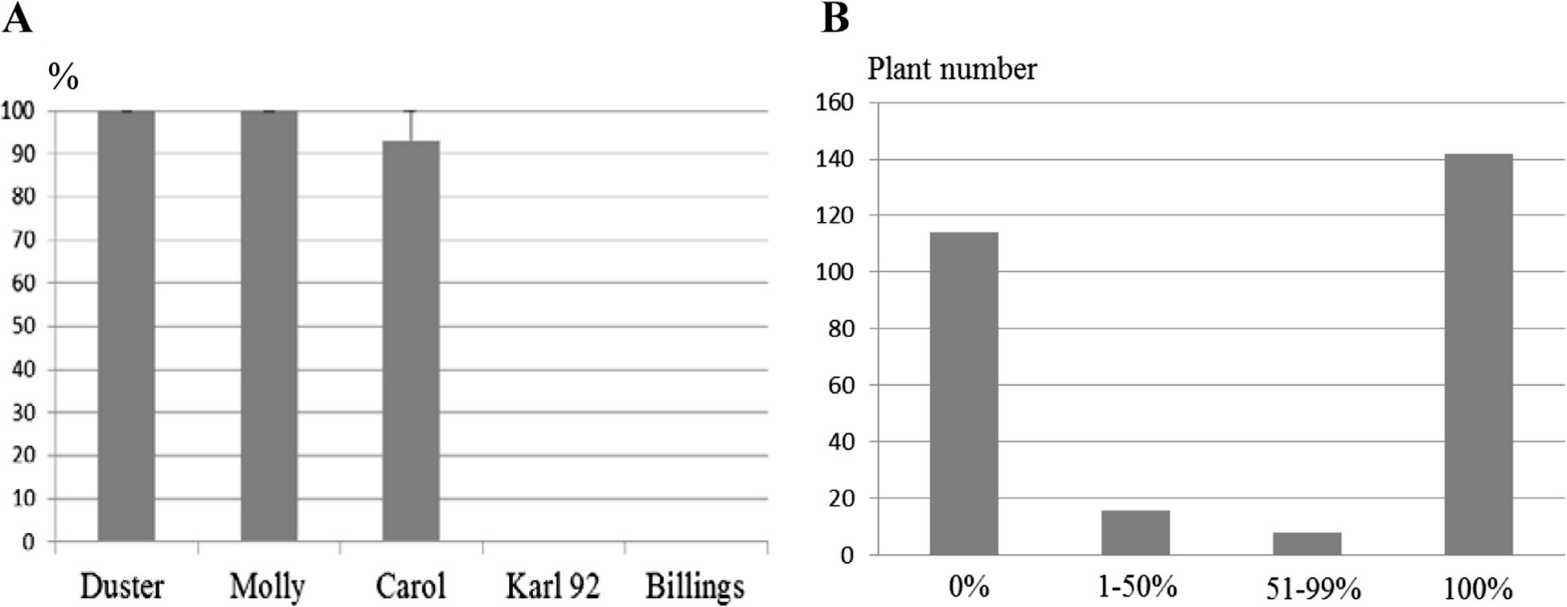
**Comparative analysis of Hessian fly resistance among hexaploid wheat cultivars. A)**. Reactions of cultivars to the biotype GP were rated (%) for comparison of Duster and Billings. Karl 92, Molly, and Carol were used as controls. **B)**. DH lines were groups based on their reactions to the biotype GP: complete resistance (100%), partial resistance (51-99%), partial susceptibility (1-50%), and complete susceptibility (0%).

Hf biotype GP was used to test two winter wheat cultivars: Duster and Billings. Two cultivars, Molly (*H13*) and Carol (*H3*), served as resistant controls, and Karl 92 (no R gene) was used as the susceptible control (Figure 1A). Like Molly, Duster showed complete resistance, whereas like Karl 92, Billings showed complete susceptibility. When 2174 was tested with the same Hf biotype, this cultivar showed approximately 70% resistance [4], indicating that Duster produces a more consistent phenotype in response to biotype GP than 2174.

The Duster x Billings population of 282 DH lines was tested for response to Biotype GP. Following infestation with the GP biotype, 142 lines showed complete resistance (similar to Duster), 114 lines showed complete susceptibility (similar to Billings), 24 lines showed intermediate resistance (16 lines with 1-50% resistance and 8 lines with 51-99% resistance) (Figure 1B), and 2 lines produced no data. When the subset of 16 intermediates was treated as susceptible and the other subset of 8 intermediates was treated as resistant, segregation of the DH population fit a one-gene model (*χ*^2^ = 1.44, *p* > 0.05).

### Genetic mapping of GBS markers in the DH population

Three GBS libraries at 96-plexing using the methods of Poland et al. [20] with enzymes *Pst* I and *Msp* I for 282 DH lines and 3 replicates of each parent. A total of 2,358 GBS markers were eventually generated from 14,028 SNP called.

The GBS SNP markers of 260 DH lines were analyzed after removing 22 lines with excessive missing data. These GBS markers were assembled into 26 linkage groups, forming genetic maps for the winter wheat DH population (Table 1). Based on the conserved locations of the GBS SNP markers, these linkage groups were assigned to 19 of the 21 chromosomes in hexaploid wheat (Table 1). Total length of the 26 linkage groups containing the 2,358 GBS markers was 2085.7 cM, with a marker density of 0.88 cM per marker. Detailed information for the length of each linkage group and genetic distances of the GBS markers on the whole genome is provided in Additional file 1: Table S2. Whereas 299 GBS markers were observed on chromosome 3B alone, no GBS markers could be mapped to chromosomes 4D or 6D. A total of 891 markers was assigned to genome A, 1,236 markers to genome B, and only 231 markers to genome D. These results supported previous observations that genome D harbors the least amount of sequence diversity [17].

**Table 1:** **Chromosomal locations of linkage groups assembled with GBS markers**

**Linkage group**	**Marker number**	Chrom.*	**Chrom. Length (cM)**	**Max. distance**	**Min. distance**	**Marker density**
4	205	1A	183.92	13.98	0	0.88
8	197	1B	120.32	18.16	0	0.61
14	61	1D	22.54	10.57	0	0.37
6	71	2A	78.06	15.3	0	1.10
24	18	2A	15.58	5.53	0	0.87
5	88	2B	38.03	7.98	0	0.42
17	50	2B	26.29	11.19	0	0.53
13	62	2D	16.14	2.28	0	0.26
23	17	2D	30.15	22.18	0	1.77
15	29	3A	71.15	19.95	0	2.45
16	11	3A	33.19	12.77	0	3.01
1	299	3B	228.36	22.18	0	0.76
21	13	3D	64.44	19.63	0	4.96
3	219	4A	212.39	15.31	0	0.97
26	15	4B	75.48	21.72	0	5.03
12	34	5A	42.69	10.35	0	1.26
22	21	5A	31.74	5.57	0	1.51
9	152	5B	214.59	23.52	0	1.41
19	34	5D	19.13	8.78	0	0.56
10	148	6A	113.35	11.61	0	0.77
7	181	6B	83.344	20.52	0	0.46
11	116	7A	110.94	13.56	0	0.96
20	19	7A	22.26	16.54	0	1.17
2	254	7B	172.74	20.94	0	0.68
18	28	7D	35.66	21.52	0	1.27
25	16	7D	23.32	9.77	0	1.46
26	2358		2085.7	23.52	0	0.88

*No GBS markers are mapped to 4D or 6D.

Although the GBS markers did not cover all chromosomes and some chromosomes were observed to have large gaps between mapped linkages, a large-effect QTL was found to account for most of the phenotypic variation in Hf biotype GP reaction of the entire population. Therefore, no further effort was needed to generate new markers to cover the missing chromosomes.

### A single gene segregated for Hf resistance in the DH population

**Figure 2:**
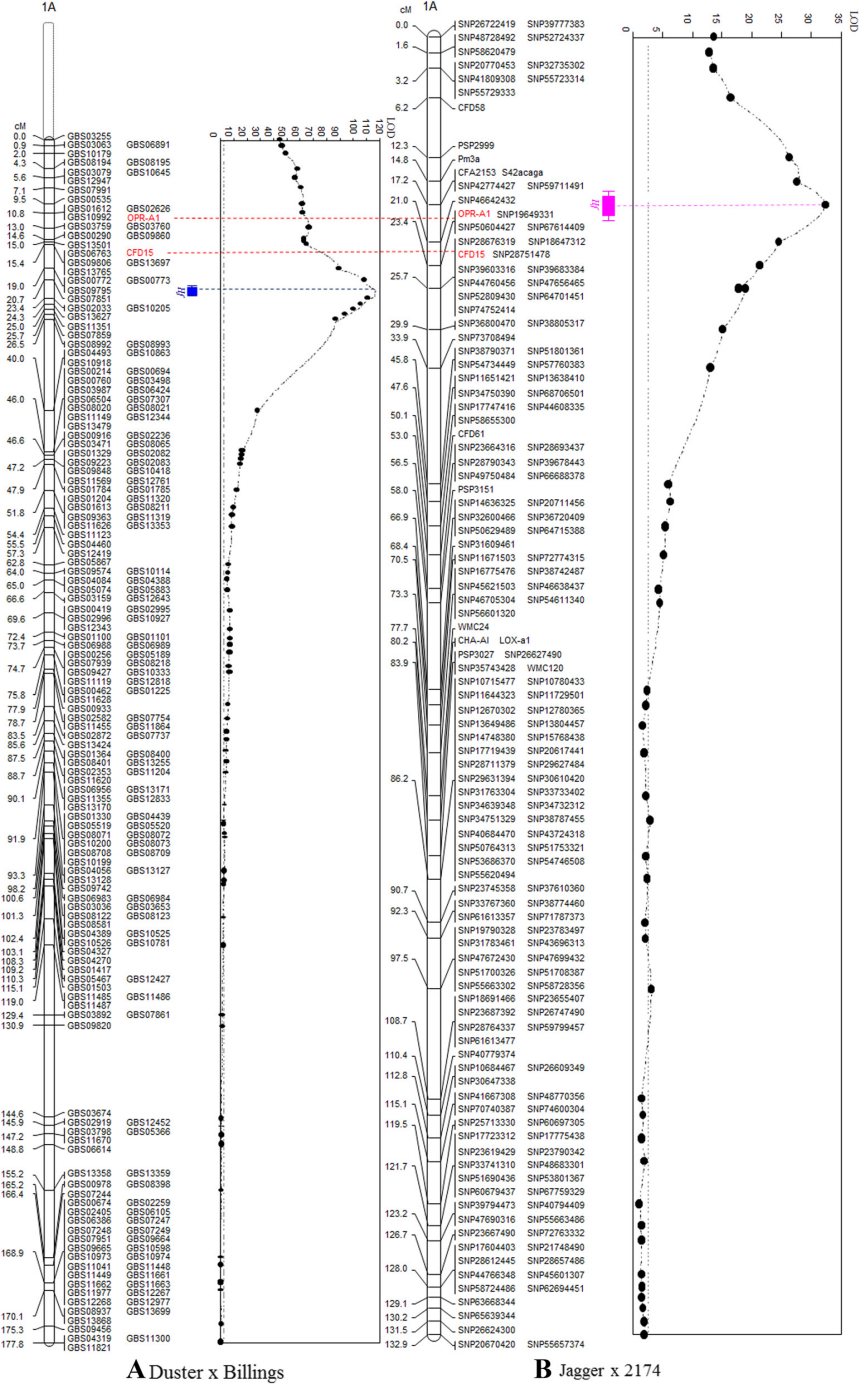
**Comparison of two QTLs for resistance to Hessian fly. A)**. The position of the *QHf.osu-1A* ^*d*^ locus in the Duster × Billings DH population was mapped using 176 GBS markers. The physical location of the QTL on the short arm of chromosome 1A was validated by using *Xcfd15* and *TaOPR-A1* markers that are highlighted in red. The gene at the *QHf.osu-1A* ^*d*^ locus is centered in a 2.6 cM region flanked by GBS07851 and GBS10205 markers that are highlighted in blue. The telomere region of chromosome 1AS that is not covered by GBS markers is indicated by a chromosomal fragment with a dotted line. **B)**. The position of the *QHf.osu-1A* ^*74*^ locus in the 144 Jagger × 2174 RILs was mapped using 154 SNP markers and 15 other markers. The physical location of the QTL on the short arm of chromosome 1A was validated by using *TaOPR-A1* and *Xcfd15* markers that are highlighted in red. The gene at the *QHf.osu-1A* ^*74*^ locus is centered in a small region indicated with purple and covering *TaOPR-A1* and *Pm3*. The vertical dotted line indicates the logarithm of the odds (LOD) significance threshold of 2.5. Common markers *TaOPR-A1* and *Xcfd15* on the two maps are aligned up to indicate their relative positions on chromosome 1AS. *QHf.osu-1A* ^*d*^ is on the proximal side of the common markers, whereas *QHf.osu-1A* ^*74*^ is on the distal side of the common markers.

A total of 205 GBS markers was assembled into linkage group 4 spanning 177.8 cM in genetic distance. The chromosomal location of the linkage group was identified by using three different approaches (below). Whereas many markers were assembled into a cluster, a 13.5 cM gap was observed between markers GBS08992 and GBS10863, which are located in the central region of the short arm of chromosome 1A (Figure 2A). Similar gaps in the same region were frequently observed in previous mapping studies [4,21].

On the basis of whole-genome QTL scanning using Interval Mapping (IM) analysis, the QTL for Hf reaction was found in this group. The LOD value at the peak position of this QTL for Hf resistance was 117, and this QTL alone accounted for up to 88% of the total phenotypic variation (Figure 2A). This is consistent with our conclusion of a bimodal phenotypic distribution for Hf reaction, which in this population was controlled by a single major gene. It was confirmed that the Duster allele confers a resistant reaction, whereas the Billings allele confers a susceptible reaction.

The intermediate phenotype in the DH population may be explained by two minor QTLs (Additional file 2: Figure S2). One QTL was mapped to linkage group 3 assigned to chromosome 4A, and this locus accounted for 7.5% of the total phenotypic variation (Additional file 2: Figure S2A). The other QTL was mapped to linkage group 11 assigned to chromosome 7A, and this locus accounted for 6.8% of the total phenotypic variation (Additional file 2: Figure S2B). At both loci Duster contained a susceptible allele and Billings contained a resistant allele. At GBS08246 on the 4A locus, the average resistance was 44% in the DH lines carrying the Duster allele but 67% in the DH lines carrying the Billings allele, indicating a significant difference in Hf resistance between the two alleles (*p* > 0.01). At GBS00103 on the 7A locus, the average resistance was 46% in the DH lines carrying the Duster allele but 66% in the DH lines carrying the Billings allele, indicating a significant difference in Hf resistance between the two alleles (*p* > 0.01).

### Validation of *QHf.osu-1A*^*d*^ on chromosome 1AS

The PCR product of a GBS marker is randomly amplified from the wheat genome; therefore, the physical location of a GBS marker is not implicit. As the present study advanced, a draft sequence of the wheat genome was recently released in the International Wheat Genome Sequencing Consortium (IWGSC) [22]. The chromosome arm-based sequences (https://urgi.versailles.inra.fr/) provided a powerful tool for identification of chromosomal locations of the GBS markers. While the location of each linkage group was predicated from the IWGSC sequences (Table 1), the physical location of the QTL on chromosome 1A was validated by three approaches.

First, the sequence of a GBS marker under the peak of the QTL was searched in IWGSC databases to determine the chromosomal location of the linkage group. As a result, the sequences of GBS markers under the QTL peak, such as GBS 07851, GBS10205, GBS02033, and GBS07859, were found identical to sequences of the contigs from chromosome 1AS. The sequences of GBS markers on the whole genome are archived in the NCBI SRA (accession number SRP051982). The sequences of those GBS markers under the QTL were also searched in EST databases in GenBank to determine if any marker hit any wheat EST that has been mapped in wheat bins [23] or any published genetic maps. If a marker did not hit any EST, the marker sequence was used to search in wheat genome sequences to test if the marker hit any contig that contains a gene. GBS07859 showed a match with contig1041452 (1,002 bp) of Chinese Spring genomic sequences (http://www.cerealsdb.uk.net) and a cDNA sequence of diploid wheat *T. urartu* (UCW_Tu-k41_contig_83), suggesting that the GBS07859 marker sequence was amplified from a gene. The sequence of GBS07859 hit a gene that has a single copy in rice chromosome 5, suggesting that the GBS07859-containing linkage group was from chromosome group 1 in wheat.

**Figure 3:**
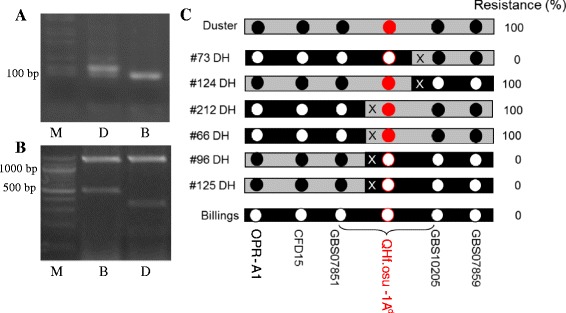
**Genotypes and phenotypes of critical recombinant lines at the** ***QHf.osu-1A*** ^***d***^ **locus in the Duster × Billings DH population. A)**. SSR marker *Xcfd15*. D is Duster, B is Billings, and M is DNA marker. **B)**. PCR marker for *TaOPR-A1*. D is Duster, B is Billings, and M is DNA marker. **C)**. Genotypes and phenotypes of six lines that have a crossover at the *QHf.osu-1A* ^*d*^ locus. X indicates a crossover between two flanking markers. A black dot represents the Duster allele, and a white dot represents the Billings allele. The gene *TaHf-A1* at the *QHf.osu-1A* ^*d*^ is predicted to be between GBS07851 and GBS10205 and indicated with a dot with red line.

Second, SSR marker *Xcfd15* that was reported on chromosome 1AS was mapped in the GBS07859 linkage group. Two SSR markers *Xcfd15* and *Xwmc432* that were mapped in chromosome group 1 were found to be polymorphic between the Duster and Billings alleles. *Xwmc432* was mapped to chromosome 1D (data not shown). *Xcfd15* was mapped to chromosome 1A (Figure 3A), further supporting that the GBS linkage group 4 was from chromosome 1A.

Lastly, a marker for the *TaOPR-A1* gene that was mapped on the short arm of chromosome 1A [4] was developed to identify allelic variation between Duster and Billings (Figure 3B). The *TaOPR-A1* gene was mapped in GBS linkage group 4 of the DH population, enabling us to conclude that the GBS linkage group 4 was located on the short arm of chromosome 1A in wheat.

The confirmed chromosomal locations of GBS07859, *Xcfd15*, and *TaOPR-A1* altogether support the location of GBS linkage group 4 on the short arm of chromosome 1A in wheat. This QTL in Duster was thus named *QHf.osu.1A*^*d*^. The previous QTL mapped in 2174 [4] is renamed *QHf.osu-1A*^*74*^.

### Two Hf resistance genes on chromosome 1AS

A total of 154 SNP markers was assembled into chromosome 1AS that was previously mapped with 15 SSR markers and PCR markers developed from genes including *Pm3*, *TaOPR-A1*, and *LOX-A1* [4]. The linkage group spanned 133 cM, with 0.87 cM per marker. The SNP-saturated map resulted in *QHf.osu-1A*^*74*^ producing an LOD value of 32.5, accounting for 66.5% of the total phenotypic variation (Figure 2B). Mapping of both the *TaOPR-A1* gene and *Xcfd15* in a set of Jagger × 2174 RILs and Duster × Billings DH lines allowed determination of the physical locations of the two QTLs. To calculate the genetic distance between the QTLs/genes, the regions containing the two QTLs and their neighboring markers were enlarged and shown in Additional file 3: Figure S1. The two common markers *Xcfd15* and *OPR-A1* are on the distal side of the peak of *QHf.osu-1A*^*d*^ in Duster but on the proximal side of the peak of *QHf.osu-1A*^*74*^ in 2174, indicating that the two Hf resistance genes are located in different regions of the same chromosome (1AS). *Xcfd15* at 15.4 cM in Duster was 5.3 cM distal to the Hf gene at 20.7 cM, but *Xcfd15* at 25.7 cM in 2174 was 4.7 cM proximal to the Hf gene at 21 cM, indicating that the two genes reside 10 cM apart. Similarly, *OPR-A1* at 10.8 cM was 9.9 cM distal to the Hf gene in Duster, but *OPR-A1* at 23.4 cM was 2.4 cM proximal to the Hf gene in 2174, suggesting that the two genes reside 12.3 cM apart. Putting the genetic distances of the two common markers together with the genes in the two different mapping populations, this study indicated that the two genes reside 11.2 cM apart. The sequence of GBS07859 representing *QHf.osu-1A*^*d*^ was identical to its rice orthologous gene at position 516 kb of chromosome 5 (300 Mb) of rice (GenBank accession number (NC_008398). *TaOPR-A1* representing *QHf.osu-1A*^*74*^ had a high identity to its rice orthologous gene at position 5,884 kb on the same chromosome 5, suggesting that the rice collinear regions of the gene at *QHf.osu-1A*^*d*^ and the gene at *QHf.osu-1A*^*74*^ spanned approximately 5,368 kb in rice.

Thorough analysis of genotypes and phenotypes of the 260 individual DH lines showed the gene responsible for the *QHf.osu.1A*^*d*^ locus resides between GBS07851 and GBS10205*.* Among the DH lines, two lines (#73 and #124) showed a crossover between GBS010205 and the gene (Figure 3C). For example, the #73 line had the Duster allele for GBS10205 but the Billings allele for GBS07851. This line was Hf susceptible, as conferred by the Billings allele. Hence, the gene allelic form in this line is the same as GBS07851. Similarly, four lines (#66, #96, #125, and #212) had a crossover between GBS07851 and the gene at *QHf.osu-1A*^*d*^ (Figure 3C). These results showed that the resistance gene at *QHf.osu-1A*^*d*^ is located in the 2.7 cM region flanked by GBS07851 and GBS10205.

## Discussion

Resistance genes against Hf have been repeatedly mapped to the end of the short arm of chromosome 1A. The previous studies suggested that this genomic region may contain a cluster of major dominant resistance genes against multiple Hf biotypes [10,13]. However, the previous studies were performed using different mapping populations, different markers, as well as different biotypes, which make it difficult to determine if a single resistance gene has multiple alleles or if several resistant genes reside on the short arm of chromosome 1A. This study demonstrates the existence of two distinct resistance genes on the short arm of chromosome 1A. The presence of the two genes in locally adapted cultivars provides more options for introgression of resistance from diverse genetic backgrounds.

Four genes (*H9*, *H10*, *H11*, and *Hdic*) on chromosome 1A were previously mapped in close linkage with SSR marker *CFA2153* at a genetic distance of less than 1 cM [10]. *H9* was also linked to *Pm3* at a genetic distance of 4.5 cM [9]; *H16* and *H17* were located at 3.7 cM and 6.2 cM to *PSP2999* in genetic distance [13]. *CFA2153*, *Pm3*, and *PSP2999* were all mapped under the peak of the *QHf.osu-1A*^*74*^ locus observed in wheat cv. 2174, suggesting that the resistance gene in 2174 could be orthologous to one or more of the previously mapped resistance genes (*H9*, *H10*, *H11*, *H16*, *H17*, and *Hdic*) in tetraploid wheat. The *TaOPR-A1* gene is the candidate for *QHf.osu-1A*^*74*^ in 2174 [4]. However, the resistance gene in 2174 and the resistance gene in Duster are at least 10 cM apart. The gene mapped in Duster is a novel one. The novel Hf resistant locus in Duster explained 88% of the phenotypic variation, suggesting that the resistance in the Duster x Billings DH population segregated according to a single gene. Most DH lines in the population showed complete resistance or complete susceptibility to Biotype GP, also supporting a one-gene model for the phenotypic distribution. Whereas the gene in cultivar 2174 explained the majority of the phenotypic variation [4], the gene in Duster produces a more consistent phenotype in response to biotype GP [14].

The presence of a major gene in the DH population with nearly unambiguous segregation of Hf resistance has provided an excellent population for cloning of this gene. To date, 14 genes have been cloned from wheat using the positional cloning strategy [24,25], but no gene has been cloned for resistance to Hf. Fine collinearity at the *QHf.osu-1A*^*74*^ locus between wheat, rice, and Brachypodium has indicated low collinearity of the gene order in this region among these species, and that the fine physical map for *QHf.osu-1A*^*74*^ cannot be established by using genome information from rice or Brachypodium only [4]. The gene in Duster has been delimited to a region between two GBS markers, GBS07851 and GBS10205. GBS07859 can be used as starting point for anchoring a physical contig of the wheat genomic sequences. The recently released genome sequences may provide a powerful tool in cloning *QHf.osu-1A*^*d*^.

GBS markers offer several advantages, including a generic sample preparation method, a highly robust genome complexity reduction strategy to facilitate *de novo* marker discovery across entire genomes, and a uniform bioinformatics workflow strategy to achieve genotyping goals tailored to individual species, regardless of the availability of a reference sequence [20]. The most distinguishing features of this technology are the ability to genotype any population structure, regardless whether parental data is included, and the ability to co-dominantly score SNP markers segregating in populations [20]. Using this new genotyping approach on biparental double haploid populations, we identified *QHf.osu-1A*^*d*^. The development and application of GBS markers in the DH population has provided a successful example of developing high-density markers in wheat without a sequenced genome, and for determining the physical location of a major gene without sequencing physical contigs or the whole genome in wheat.

Among the 35 known Hf resistance genes, however, only 8 genes (*H1-H5*, *H7*, *H8*, and *H12*) were identified in hexaploid wheat [26,27]. The remaining 25 genes were identified in distant and close relatives of hexaploid wheat. *H9*, *H10*, and *H11* were individually transferred from *T. turgidum* ssp. *durum* into the background of common wheat cultivars [28], but these genes have not yet been deployed in commercial cultivars [10,29]. *Hdic* was transferred to wheat germplasm KS99WGRC42 from an accession of cultivated emmer wheat (*T. turgidum ssp. dicoccum*) PI 94641. It is not known where the gene in 2174 or Duster originated. However, this study provides molecular marker tools to pyramid effective resistance genes in bread wheat, particularly those accessible from more adapted genetic backgrounds, to manage Hessian fly and improve resistance durability in hard red winter wheat.

## Conclusion

This study deployed GBS markers to rapidly and precisely map a major gene for unique resistance against Hf in winter wheat cultivar Duster. In comparison with the Hf resistance gene in winter wheat cultivar 2174, the gene in Duster is novel and confers a more consistent phenotype. The Hf resistance gene in 2174 is likely allelic to one or more of the previously mapped resistance genes (*H9*, *H10*, *H11*, *H16*, or *H17*) in wheat, but the Hf resistance gene in Duster is not allelic to any of these reported genes. The existence of two distinct resistance genes on the short arm of chromosome 1A in locally adapted cultivars provides more options for introgression of resistance from diverse genetic backgrounds and pyramiding of the distinct two resistance genes in a single germplasm line. The nearly unambiguous segregation of Hf resistance under Duster and Billings genetic backgrounds has provided an excellent opportunity for cloning of the Hf resistance gene in Duster.

## Methods

### Hessian fly biotype and resistance of the plants to Hessian fly

Hf biotype GP was used in this study. Biotype GP was maintained in the USDA-ARS Hard Winter Wheat Genetics Research Unit, Manhattan, Kansas, USA. Biotype GP used in this study. The Hf population was maintained in the greenhouse with wheat seedlings of ‘Karl 92’ , which is 100% susceptible to biotype GP.

Duster and Billings are two winter wheat cultivars that were released in the southern Great Plains, and the two hard red wheat cultivars were used to generate a population with 282 doubled haploid (DH) lines. Jagger and 2174 are also two winter wheat cultivars utilized in in the southern Great Plains, and the two hard red wheat cultivars were used to generate a population of recombinant inbred lines that were tested for response to Biotype GP in a previous study [4]. The infestation experiment with Hf on parental lines and 282 DH lines of Duster × Billings were conducted using the approach as described previously [4]. The reaction of a line was recorded as susceptibility or resistance. When all plants of a line were susceptible, this line was phenotyped as 0% resistance. When all plants of a line were resistant, this line was phenotyped as 100% resistance. A random subset of the DH lines was confirmed with three replications.

### Development of GBS markers

Three 96-plex libraries were generated from a single sample of each DH line and three replicates of each parent. A library consisting of DNA fragments with a forward adapter and a reverse adapter on opposite ends of every fragment was generated for each of the DH lines according to the protocols of Poland et al. [20] using restriction enzymes *Pst* I and *Msp* I to produce a complexity reduction of the genome and capture the genomic sequence between restriction sites. The full list of barcoded adapters and the list of DH samples with corresponding barcodes are provided in Additional file 4: Table S1. The procedures of library construction were described previously [20].

PCR products were amplified using a program with a short extension time (<30 s) to enrich shorter fragments suitable for bridge-amplification on the Illumina flow-cell on Illumina HiSeq2000. The raw sequences were assigned to individual samples base on an exact match to the DNA barcode followed by the *Pst* I restriction site and trimmed to 64 bp. The tag sequences were aligned allowing a one or two base-pair difference to call putative SNPs. When a SNP call showed a difference between the two alleles, the SNP was considered as a GBS marker. If a SNP call was heterozygous, presumably due to sequencing errors, this call was set to missing data. To ensure linkage map quality, the SNP markers used for final mapping were selected by removing markers that were more than 20% missing values and contained identical recombinant information. Eventually, a total of 2,358 GBS markers were generated from 14,028 SNP called.

### Linkage group construction and QTL analysis

The 2,358 GBS markers developed for the Duster x Billiongs population were analyzed for linkage mapping using the 260 DH lines. To more precisely map the gene responsible for *QHf.osu-1A*^*74*^ in 2174, SNP markers were used to saturate the targeted region in 144 Jagger × 2174 recombinant inbred lines (RILs). The SNP markers were generated by an Illumina Infinium 9 K iSelect platform through the TCAP [17], and the 8-digit SNP codes served as the reference number for each SNP.

The GBS and SNP markers were used to make linkage groups using JoinMap 4.0 [30]. The Kosambi mapping function was used to estimate the map distance. The Interval Mapping program was run to locate QTLs for the Hf resistance using MapQTL 6.0 [31]. Logarithm of the Odds (LOD) threshold for significance was 2.5 for the presence of a putative QTL. Maximum LOD values were used to estimate QTL peak positions.

### Development and mapping of SSR markers and *TaOPR-A1* genes

Forward primer 5'-CTCCCGTATTGAGCAGGAAG-3' and reverse primer 5'-GGCAGGTGTGGTGATGATCT-3' were used to amplify products for *Xcfd15*. The PCR amplification was performed as follows: 94°C for 3 min, 40 cycles of 94°C for 30 sec, 55°C for 30 sec, and then 72°C for 30 sec, the final extension was 72°C for 10 min. The PCR products were separated and scored on 2% agarose gel.

There are four copies of *TaOPR-A1* genes in a BAC clone of *T. durum* that were mapped associated with the *QHf.osu-1A*^*74*^ found in 2174. Two primers, OPR1A4-F1 (5’-TCACTACCACACCACTCAG-3’) and OPR1A4-R1 (5’-CATCTAATTAGTGTCCTGCA-3’) were designed to amplify the forth copy of *TaOPR-A1* with 1,717 bp in size (GenBank KF035081). A SNP was found to distinguish between the Duster and Billings alleles after the PCR products were digested with restriction enzyme *BssH* II. The PCR was performed using the procedure as follows: 94°C for 3 min, 40 cycles of 94°C for 30 sec, 55°C for 30 sec, and then 72°C for 2 min, the final extension was 72°C for 10 min. The PCR products were purified and sequenced. The digested PCR products were 143 bp, 324 bp, and 1,250 bp for the Duster allele but 467 bp and 1,250 bp for the Billings allele. The marker developed for *TaOPR-A1* was used to analyze the DH population.

## Additional files

Additional file 1: Table S2. The chromosomal locations and linkage groups of GBS markers. Additional file 2: Figure S2. Mapping of two minor QTLs for resistance to Hessian fly. Additional file 3: Figure S1. The enlarged regions containing the two QTLs and their neighboring markers. Additional file 4: Table S1. Barcode adapters used for GBS.

## Abbreviations

GBS: Genotyping-by-sequencing
SNP: Single-nucleotide polymorphism
QTL: Quantitative trait loci
Hf: Hessian fly
DH: Doubled haploid
SSR: Single sequence repeat
cM: Centimorgan
NGS: Next-generation sequencing
IM: Interval Mapping
LOD: Logarithm of the Odds
PCR: Polymerase chain reaction
EST: Expressed sequence tags
RIL: Recombinant inbred line
kb: Kilo base pair
bp: Base pair
