# Supplementary material for: Precisely mapping a major gene conferring resistance to Hessian fly in bread wheat using genotyping-by-sequencing
Source: BMC Genomics. 2015 Feb 21;16(1):108. doi: 10.1186/s12864-015-1297-7 (PMC4347651; doi:10.1186/s12864-015-1297-7)
Supplement: Additional file 2: Figure S2. — Mapping of two minor QTLs for resistance to Hessian fly. [file 12864_2015_1297_MOESM2_ESM.docx]

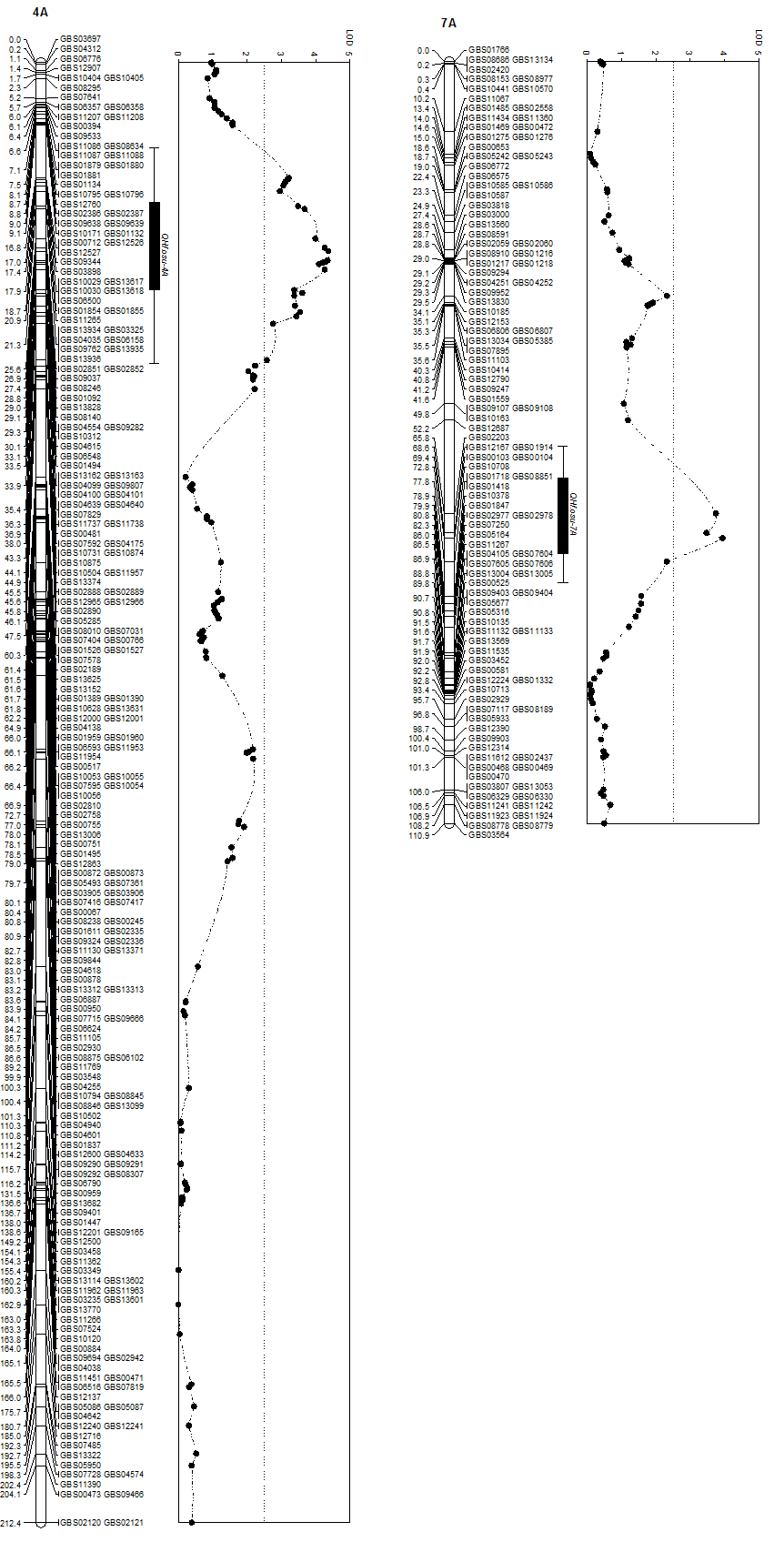
**Fig. S2.**

B

A

**Fig.S2. Two minor QTLs for resistance to Hessian fly.** A). *QHf.osu-4A*. *QHf.osu-4A* was mapped in the Duster × Billings DH population using 219 GBS markers. This QTL is centered in a 34.9 cM region flanked by GBS10171 and GBS10504. B). *QHf.osu-7A. QHf.osu-7A* was mapped in the Duster × Billings DH population was mapped using 116 SNP markers. This QTL is centered in a 25.6 cM region flanked by GBS12687 and GBS01718. The vertical dotted line indicates the LOD threshold of 2.5.
