# Supplementary material for: Precisely mapping a major gene conferring resistance to Hessian fly in bread wheat using genotyping-by-sequencing
Source: BMC Genomics. 2015 Feb 21;16(1):108. doi: 10.1186/s12864-015-1297-7 (PMC4347651; doi:10.1186/s12864-015-1297-7)
Supplement: Additional file 3: Figure S1. — The enlarged regions containing the two QTLs and their neighboring markers. [file 12864_2015_1297_MOESM3_ESM.docx]

**Fig.S1**

**A** Duster x Billings

**B** Jagger x 2174


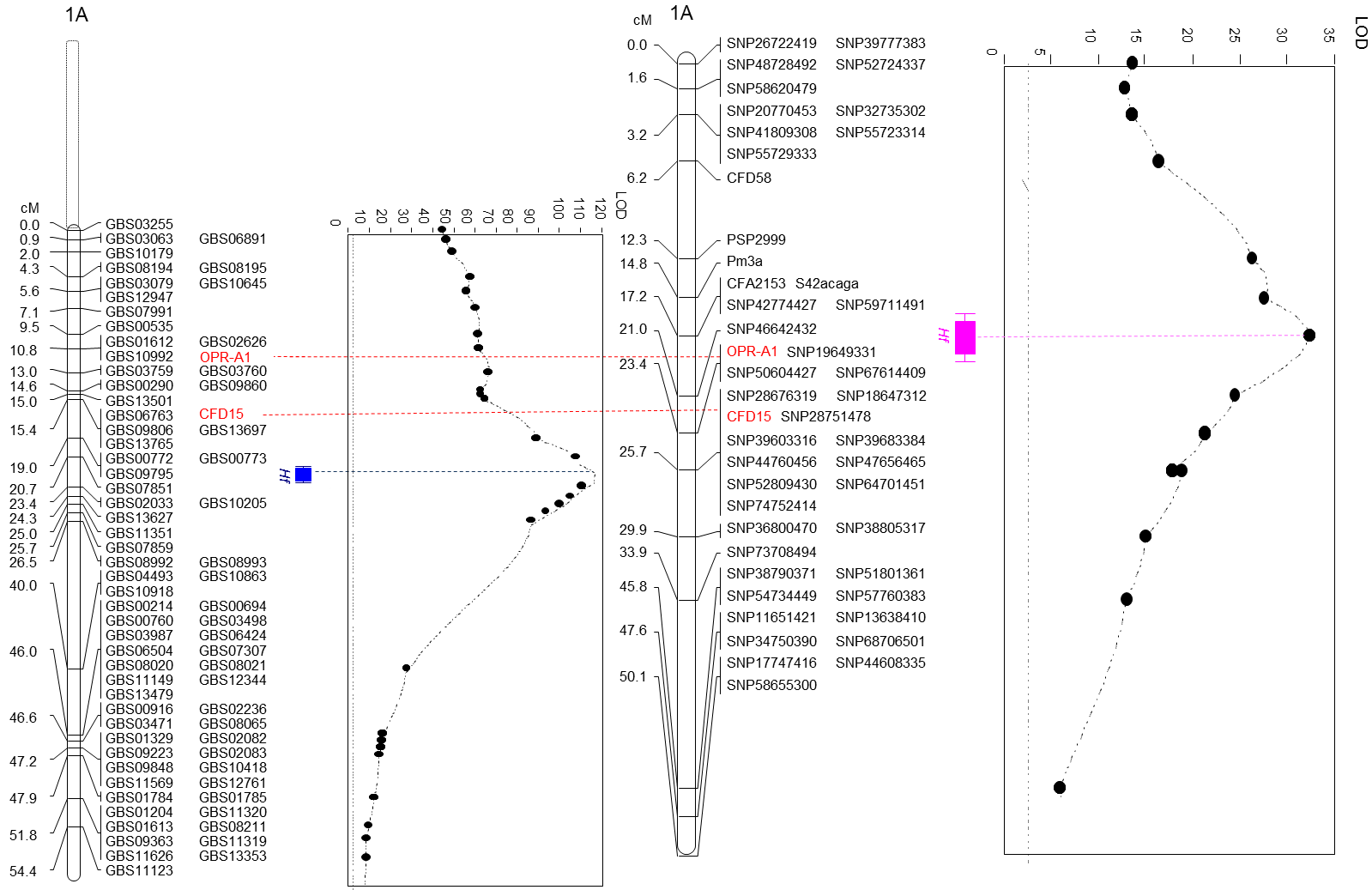


**Fig. S1. Comparison of two QTLs for resistance to Hessian fly.** The part of Fig.2 was enlarged to show the regions containing the two QTLs and their neighboring markers.
